# 

**Published:** 1889-05

**Authors:** 


### CONTENTS—MAY, 1889.—VOL. 4, NO. 11.

ORIGINAL COMMUNICATIONS— Page.

Diphtheria: by Jno. Preston, M. D., Austin, Texas, President Travis Co. Medical Society, etc. . . . . . . . . . 435
Lime in the Urine: by Prof. Odo Betz, Heilbronn, Germany 438

SOCIETY NOTES—

Proceedings Central Texas Medical Society . . . . . . . 440
Proceedings Texas State Medical Association . . . . . . 442

EDITORIAL—

The Late Meeting . . . . . . . . . . . . . . . . . . 481
R. M. Swearingen, M. D., President Texas State Medical Association. Biography . . . . . . . . . . . . . . . 486
Contemporary Physicians of Texas . . . . . . . . . . . 489

MEDICAL NEWS AND MISCELLANY—

Numerous items of interest . . . . . . . . . . . . . . 490

PUBLISHER'S NOTICES.—

Important to the profession and others . . . . . . . . . 494

